# Uncovering the transcriptome-wide RNA modifications in Acinetobacter baumannii

**DOI:** 10.1099/mgen.0.001327

**Published:** 2024-11-20

**Authors:** Kah Ern Ten, Sadequr Rahman, Hock Siew Tan

**Affiliations:** 1 School of Science, Monash University Malaysia, Bandar Sunway, Selangor Darul Ehsan, Malaysia

**Keywords:** *Acinetobacter baumannii*, direct RNA sequencing, RNA modifications, infection, *Galleria mellonella*

## Abstract

Despite being a major human pathogen, limited studies have reported RNA modifications in *Acinetobacter baumannii*. These post-transcriptional modifications play crucial regulatory roles in bacteria and have also been shown to modulate bacterial virulence. Using nanopore sequencing, we characterized RNA modifications in a virulent *A. baumannii* strain (Ab-C98) under free-living (mid-exponential phase *in vitro* culture) and during an early stage of infection (3 h post-infection) in *Galleria mellonella* larvae. Analysis revealed that m^5^C methylations are essential for ribosome synthesis, while m^6^A and Ψ are involved in metabolic pathways and translation processes. Iron-chelating genes *exbD* (m^5^C and m^6^A) and *feoB* (m^6^A and Ψ) and RNA polymerase subunit *rpoC* (m^6^A and Ψ) were selectively modified during infection. This first transcriptome-wide study highlights the potential regulatory roles of m^5^C, m^6^A and Ψ modifications in *A. baumannii* during infection.

**Impact Statement**

Post-transcriptional modifications on RNA have shown significant roles in modulating bacterial virulence. However, this has not been studied in the critical pathogen *Acinetobacter baumannii* in an *in vivo-*like model. To address this, we explored the RNA modifications (m^5^C, m^6^A and Ψ) of a virulent community-acquired *A. baumannii* via direct RNA sequencing followed by bioinformatic analysis. This study identified the locations of the RNA modifications and discussed their roles in *A. baumannii* biological pathways. Furthermore, we compared the differences in modified genes between free-living state and infection conditions. We discussed the roles of RNA modifications in supporting *A. baumannii* survival in the host during infection in *Galleria mellonella*. Our findings provide valuable references for future research on understanding the virulence roles of RNA modifications in * A. baumannii*, which could be an alternative for developing novel therapeutic agents against this pathogen.

## Data Summary

All nanopore sequencing data are available at the Sequence Read Archive (SRA, https://www.ncbi.nlm.nih.gov/sra) under the project accession number PRJNA1128375.

## Introduction

RNA consists of four nts (adenine, guanine, cytosine and uracil), and there are >160 chemical modifications reported in the RNA molecules that play various roles in regulating gene expression [1]. However, only ~60 tRNA and rRNA modifications are known in bacteria, generally categorized into three major groups: methylations, pseudouridylation (Ψ) and A-to-I editing. Ψ is one of the most abundant post-transcriptionally modified nts in various RNAs regulating important biological functions, such as stabilization of RNA conformation and translation decoding [2]. The roles of tRNA modifications for bacterial virulence and infection have been reviewed in Koh and Sarin [3]. These modifications facilitate bacterial adaptation to physiological and cellular stresses, regulating cellular responses and controlling bacterial viability and virulence during infection in a host. One of the examples is Ψ55, catalysed by the pseudouridine synthase TruB, which is critical for the regulation of multiple virulence-associated genes in the human pathogen *Shigella flexneri* and is understood to act by improving the translational efficiency of the poorly decoded CGA codon [4]. However, these studies are limited to the abundant types of RNA (rRNA and tRNA). The knowledge of RNA modifications in other types of RNA, such as mRNA, in bacterial virulence remains relatively scarce.

Previously, the detection of RNA modification required customized protocols and was usually limited to certain modifications. For example, modified nts were detected by TLC or liquid chromatography-coupled mass spectrometry (LC–MS) upon digestion of RNA molecules into mononucleotides or dinucleotide monophosphates [1]. These techniques, however, required a large amount of pure, homogenous RNA input, limiting the detection of RNA modifications to the more abundant RNAs. The advancements in the next-generation sequencing techniques improved the detection of transcriptome-wide modified RNA molecules at single-nt resolution upon chemical labelling, immunoprecipitation, enzymatic treatments or direct sequencing technology [5]. This greatly improved the sensitivity of RNA modification detection transcriptome-wide. The direct RNA sequencing established by the Oxford Nanopore omits the reverse transcription and amplification processes before the sequencing, allowing the continuous sequencing of full-length, native RNA molecules, retaining the information of the chemical RNA modifications on the RNA [6]. This sequencing technology represents a major advancement in detecting RNA modifications across the entire transcriptome without restrictions on the types of modifications. The chemical modifications can be identified and located by downstream bioinformatics tools based on the changes in the current signal generated by the nanopores between modified and non-modified RNA.

Bacterial RNA modifications have been extensively studied in the model strain *Escherichia coli* strain K-12. Recently, Jordan *et al.* [7] explored the role of the queuosine tRNA modification in *Acinetobacter baumannii* during calprotectin-mediated metal starvation. m^7^G modification on *A. baumannii* 16S rRNA (catalysed by RsmG) plays a role in bacterial susceptibility to streptomycin [8], while m^7^G tRNA modification in *A. baumannii* (catalysed by TrmB) has been shown to play a critical role in bacterial pathogenesis [9]. In this study, we analysed three types of RNA modifications [5-methylcytosine (m^5^C), *N*^6^-methyladenosine (m^6^A) and pseudouridine (Ψ)] in the community-acquired *A. baumannii* strain C98 (Ab-C98) that was isolated from the healthy community of Segamat (Johor, Malaysia) [10,11] via direct RNA sequencing. We previously reported the high virulence of Ab-C98 in the *Galleria mellonella* infection model due to the significant differential expression of genes involved in iron uptake and siderophore production [12]. However, we did not observe genes encoding RNA-modifying enzymes being differentially expressed via the transcriptomic analysis. Through the RNA modification analysis, we reported the presence of RNA modifications on protein-encoding genes, rRNA and tRNA and their potential roles in Ab-C98 at an early stage of infection (3 h post-infection) in the *G. mellonella* larvae. These modifications could play a significant role in facilitating bacterial survival during the infection.

## Methods

The biological RNA samples of Ab-C98 were prepared and sequenced (direct RNA-seq) previously [12]. Briefly, total RNA of free-living Ab-C98 (IVB) was isolated from the mid-exponential phase of bacterial culture grown in an LB medium. For the infection samples (IVV), the bacterial cells were isolated and enriched from the infected *G. mellonella* larvae (sixth instar stage) at 3 h post-infection, and the total RNA was extracted. The bacterial RNA samples were then sequenced on a MinION sequencer for direct RNA-seq. Here, we described the methodology used to construct non-modified negative control (*in vitro*-transcribed RNA) and the RNA modification analysis from the nanopore sequencing data generated from the direct RNA-seq via different bioinformatics tools.

### *Invitro* transcription control RNA preparation

A negative control library [*in vitro* transcription control (IVT)] utterly devoid of RNA modification was used to remove the false positives due to the sequencing background noise. To construct this library, the poly(A)-tailed total RNA of Ab-C98 was dephosphorylated by RNA 5'-pyrophosphohydrolase (RppH) (NEB, M0356S). The RNAs with 5’-monophosphate were ligated to the T7 promoter using T4 RNA ligase 1 (NEB, M0204S). Then, first-strand cDNA was synthesized using oligo d(T)_23_VN and reverse transcribed using ProtoScript® II Reverse Transcriptase (NEB, M0368S). The RNA template was removed by RNase H (NEB, M0297S), and LongAmp® Taq DNA Polymerase amplified the single-stranded cDNA to generate a library of double-stranded DNA (dsDNA). Then, the dsDNAs were used as the template for *in vitro* transcription using HiScribe® T7 Quick High Yield RNA Synthesis Kit (NEB, E2050S) with DNase I treatment according to the kit’s manual. After purification, a second in-tube DNase I treatment (Monarch DNase I) was performed at 37 ℃ for 1 h. Complete removal of DNA templates was verified by PCR amplification of the housekeeping gene *rpoD*. RNA samples with no PCR products generated indicate the successful elimination of gDNA. The *in vitro*-transcribed RNAs were then poly-adenylated by *E. coli* Poly(A) polymerase (NEB, M0276S). The presence of poly(A)-tails was confirmed by reverse transcription using oligo d(T)_23_VN (NEB) to generate RNA–cDNA hybrids and PCR amplification of the housekeeping gene *rpoD*. Only RNAs with polyA-tail will form RNA–cDNA hybrids, allowing the *Taq* polymerase to bind to the cDNA strand, generating PCR products (183 bp). The poly(A)-tailed IVT control sequencing library was then loaded onto the R9.4.1 flow cell (FLO-MIN106) and sequenced in the MinION device (Oxford Nanopore) as described in Ten *et al*. [12].

The raw nanopore reads of the two biological replicates of the biological RNA samples (no-infection, IVB, and infection, IVV) were merged and downsampled to a similar number of reads for improved coverage and direct comparison for the RNA modification analysis, according to Huang *et al.* [13]. This is supported by the strong correlation between the two biological replicates, as reported in our earlier study [12].

### m^5^C modification analysis

The raw fast5 files were converted into single fast5 via ont_fast5_api multi_to_single_fast5 (https://github.com/nanoporetech/ont_fast5_api.git). The reads were resquiggled, and the m^5^C in each IVV and IVB was detected via the Tombo function Specific Alternate Base Detection [14] against the IVT control. The estimated fraction of modified reads at each valid reference site was generated. As the reported modification sites by each software could contain false-positive sites [15], we applied stringent criteria to generate reliable modification profiles to ensure reliability. The m^5^C positions with an estimated fraction alternate ≥0.9 were considered m^5^C-modified. Functional enrichment analysis was performed by STRING version 12.0 [16]. A *P*-value <0.05 was considered significantly enriched.

### m^6^A modification analysis

Raw nanopore sequencing fast5 files were base-called using Guppy version 3.6.1 against the m^6^A base-calling model [17], with the --fast5_out option. The base-called fast5 containing the m^6^A information was fed into ModPhred [18] for m^6^A detection and annotation. The output table generated by ModPhred consists of all the m^6^A sites found with at least 25 reads of coverage and at least 5% modification frequency in the sample, with information about coverage, modification probability, modification frequency and base-calling accuracy. The overlapped m^6^A between IVB and IVT control and IVV and IVT samples were removed to avoid false positives due to background noise. *De novo* motif discovery of the annotated m^6^A sites was performed by MEME Suite [19]. Functional enrichment analysis was performed by STRING version 12.0 [16]. A *P*-value <0.05 was considered significantly enriched.

### Pseudouridine modification analysis

The Ψ was predicted by the bioinformatics tool NanoPsU [13]. Briefly, the reads were mapped to Ab-C98 using minimap2 built-in in the NanoPsU tool. Spliced reads were removed, and the features of all U sites were extracted. Similarly, to ensure the reliability of the reported Ψ modification sites, the candidate uridines suggested by the NanoPsU with probability ≥0.9 were considered Ψ sites. To remove false positives due to the nanopore background noise, the predicted Ψ sites in the IVB and IVV were compared individually to the IVT control, and the overlapped Ψ with IVT were removed. The resulting Ψ was further compared between the IVV and IVB treatment groups to obtain Ψ unique in the IVV sample. Ψ predicted on the incorrect strand (due to the whole-genome sequence used as a reference genome) and/or target position that did not contain a ‘U’ was filtered. Functional enrichment analysis was performed by STRING [16]. A *P*-value <0.05 was considered significantly enriched. The effect of amino acid change at the Ψ-modified codon (Ψ, replaced by A/G/C) in the IVV was assessed by the web-server tool Sorting Intolerant from Tolerant (SIFT) [20].

### Validation of the presence of Ψ on Ab-C98 mRNA

The primers used for the N-cyclohexyl-N′-β-(4-methylmorpholinium)ethylcarbodiimide (CMC) -assisted RT-qPCR (reverse transcription quantitative PCR) were designed according to Dai *et al.* [21]. For each selected Ψ-modified target, two sets of RT-qPCR primers were designed for (1) Ψ-region: the 200-nt region centred by the target Ψ site; (2) control-region: the 200-nt region within this mRNA, without overlapping with the Ψ-containing 200-nt region. Ab-C98 total RNA with confirmed gDNA removed was subjected to CMC treatment, according to Zhang *et al.* [22], with slight modifications. Briefly, the RNA was denatured at 65 °C for 15 min. CMC at a concentration of 1 M (Sigma, C106402) was prepared freshly in TEU buffer [50 mM Tris-HCl (pH 8.3), 4 mM EDTA, 7 M urea]. One thousand nanograms of denatured RNA in 12 µl was mixed with 4 µl of 1 M CMC buffer, 24 µl of TEU buffer and 40 units of RNase inhibitor (NEB) as the ‘+CMC’ sample. For the ‘-CMC sample’, 1000 ng of denatured RNA in 12 µl was mixed with 28 µl TEU buffer and 40 units of RNase inhibitor. The mixture was incubated at 30 °C for 16 h. The RNA was then precipitated at −80 °C overnight by adding 140 µl of RNase-free water, 20 µl of sodium acetate and 550 µl of absolute ethanol. The recovered ‘+CMC’ and ‘-CMC’ RNA were then separately resuspended in 40 µl Na_2_CO_3_ buffer and 20 units of RNase inhibitor and incubated at 37 °C for 6 h. This step selectively labels the Ψ by removing CMC adducts on Us and Gs while leaving CMC-Ψ mono-adduct on the RNA. An additional ethanol precipitation step was performed. The recovered RNA was dissolved in 12 µl nuclease-free water. The reverse transcription was conducted on 50 ng of the ‘+CMC’ and ‘-CMC’ RNA samples with gene-specific primer (Ψ-region and control-region) using 50 units of ProtoScript II reverse transcriptase (NEB) in 20 µl reaction. Luna® Universal qPCR Master Mix (NEB) and CFX Duet (Bio-Rad) were used for RT-qPCR quantitation with the following conditions: 95 °C for 1 min, followed by 40 cycles of 95 °C for 15 s and 60 °C for 30 s. A melt curve analysis was performed at the end of each qPCR to analyse the specificity of the qPCR primers. The known Ψ516 on the *rrsA* gene encoding 16S rRNA of *E. coli* was used as a reference [23], using strain DH5α, an *E. coli* K-12 derivative [24], as the reference bacterial model. The relative Ψ level was calculated by first obtaining the ΔCq(-) = Cq(Ψ-region, -CMC) - Cq(control-region, -CMC) and ΔCq(+) = Cq(Ψ-region, +CMC) - Cq(control-region, +CMC) and then, ΔΔCq(Ψ-region) = ΔCq(+) - ΔCq(-). The relative Ψ level is represented as 2^ΔΔCq(Ψ-region)^. Student t-test was performed to analyse the statistical difference in the relative Ψ levels between the infection and no-infection control by Prism 10 (Version 10.2.0). A *P*-value of <0.05 was considered statistically significant. A semi-qPCR method was used for the targets when strong primer dimers were formed during the SYBR-green real-time qPCR. Briefly, the 5 ng of cDNA was used for the PCR amplification, omitting the final extension step. The PCR products were collected at 20th, 25th and 30th cycles. The same volume of PCR products was then visualized on 2% agarose gel.

## Results and discussion

To characterize the RNA modifications in *A. baumannii* in free-living, IVB, and IVV conditions, we analysed RNA modifications on the nanopore sequencing data of Ab-C98 from our earlier study [12]. As a negative control, the transcriptome of Ab-C98 was *in vitro* transcribed to construct a non-modified RNA library (IVT) for subsequent RNA modification analysis to reduce the false positives from the sequencing background noise. The nanopore sequencing of IVT control generated 594072 reads on a single R9.4.1 flow cell. Low-quality reads were trimmed, resulting in a total of 579385 reads with a median read quality of 11.3 and a median read length of 770 bp. Here, we presented the results of the m^5^C, m^6^A and Ψ predicted via bioinformatics analysis and their potential roles in *A. baumannii* and during infection in the *G. mellonella* larvae.

### m^5^C methylations are essential in the ribosome synthesis and assembly in *A. baumannii*

**Fig. 1.**
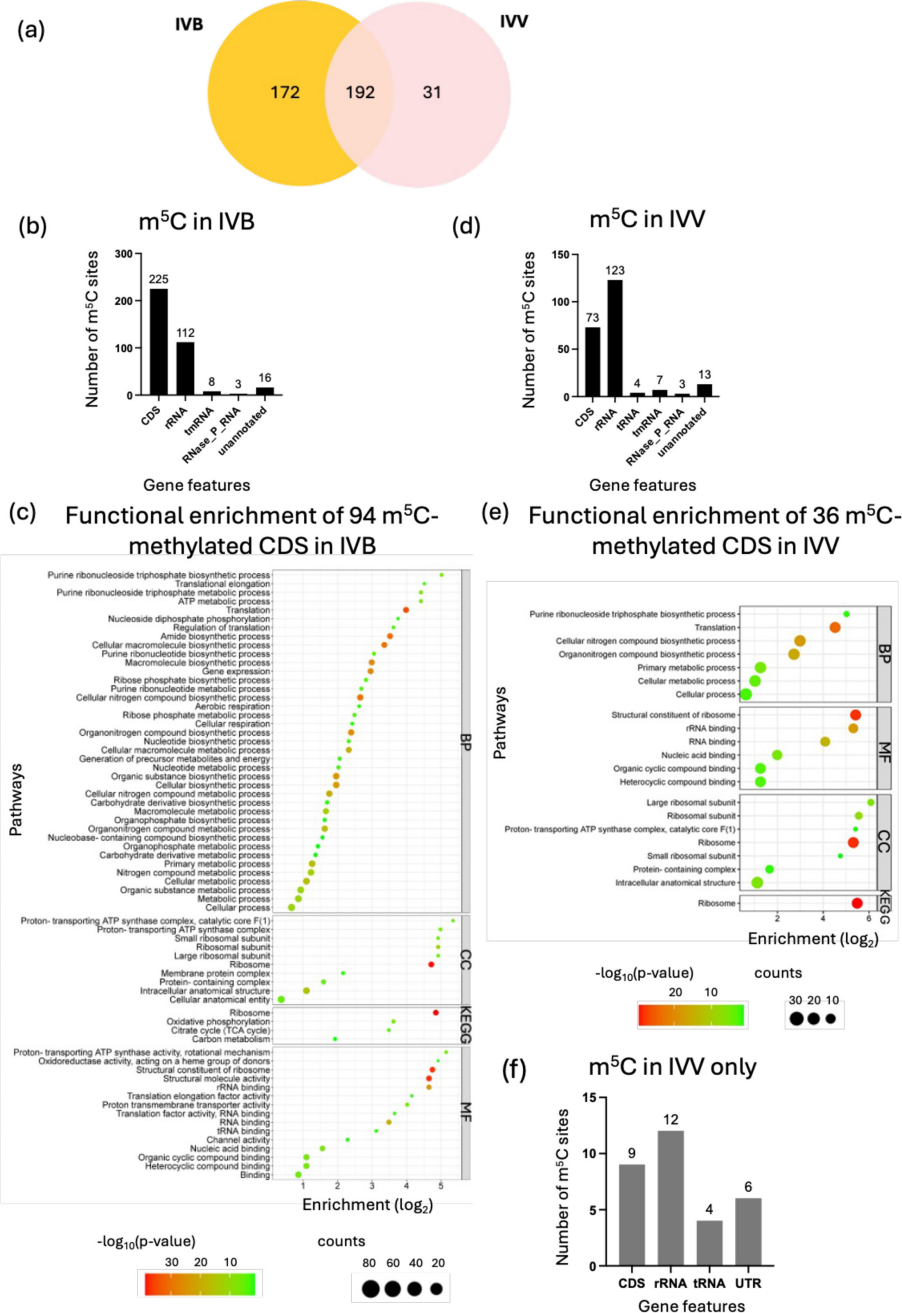
m^5^C modification analysis in Ab-C98 RNA. (**a**) Venn diagram of m^5^C modification detected in IVB and IVV. (**b**) Gene features of the gene with predicted m^5^C in Ab-C98 IVB. (**c**) Functional enrichment analysis of m^5^C-modified CDS in Ab-C98 IVB samples. (**d**) Gene features of the gene with predicted m^5^C in Ab-C98 IVV. (**e**) Functional enrichment analysis of m^5^C-modified CDS in Ab-C98 IVV. BP=Gene Ontology Biological Process, CC=Gene Ontology Cellular Components, MF=Gene Ontology Molecular Function, KEGG=Kyoto Encyclopaedia of Genes and Genomes. (**f**) Gene features of m^5^C locations were found uniquely in Ab-C98 IVV.

m^5^C is more extensively studied in eukaryotes on its occurrence in DNA, while less attention has been paid to its presence in RNA. In bacteria, m^5^C has only been described in bacterial rRNA, in which the m^5^C modifications were reported in *E. coli* 16S and 23S rRNA [25] and were not found on bacterial tRNA and mRNA yet [26]. In this study, a total of 364 and 223 candidate m^5^C sites were detected in the no-infection controls (IVB) and infection samples (IVV), respectively (Fig. 1a). In the IVB, five m^5^C-modified sites were consistently detected on the six 16S rRNA copies: m^5^C97, m^5^C931, m^5^C1054, m^5^C1223 and m^5^C1434 (Table S1, available in the online version of this article). For 23S rRNA, 12 m^5^C modifications were consistently detected (m^5^C988, m^5^C1149, m^5^C1652, m^5^C1743, m^5^C1757, m^5^C1789, m^5^C2014, m^5^C2073, m^5^C2126, m^5^C2289, m^5^C2719 and m^5^C2859) on the six 23S rRNA copies, with four of the 23S rRNA copies (NNO094_00020, NNO94_14785, NNO94_17230 and NNO94_17500) had additional m^5^C signals detected (Table S1). Tombo detected a higher number of m^5^C methylations on Ab-C98 rRNA compared to the reported m^5^C in *E. coli* rRNA (m^5^C967 and m^5^C1407 on 16S rRNA and m^5^C1962 on 23S rRNA) [25]. These m^5^C-modified sites in Ab-C98 rRNA have different position numbers than that of *E. coli*, which could be due to the difference in the 16S and 23S rRNA gene sequences of the two species (blastn=85.79 and 84.82%, respectively).

The m^5^C-modified transcripts in the IVB are mostly annotated within the ORF of the coding sequence (CDS), followed by the rRNA (Fig. 1b). This contrasts the previous reports where no m^5^C was found in *E. coli* and *Bacillus subtilis* mRNA via bisulphite-seq [27]. Of the 94 m^5^C-modified CDS in the no-infection, 39 genes were involved in the biosynthesis of ribosomal proteins (*rpl*, *rps* and *rpm* genes), representing the major cluster (Table S2). Functional enrichment analysis showed that 64 GO (Gene Ontology) terms and 4 KEGG pathways were enriched (Fig. 1c). The KEGG enrichment analysis demonstrated that the m^5^C-modified transcripts were enriched in the ribosome pathway (synthesis and assembly), whose process is energy-dependent [28]. Thus, oxidative phosphorylation, citrate cycle (TCA cycle) and carbon metabolism pathways were also enriched to generate the energy required (Fig. 1c). In contrast, rRNA has the most annotated m^5^C methylations in the infection samples (IVV), followed by the CDS that involved 36 m^5^C-modified transcripts (Fig. 1d). Functional enrichment analysis showed that 20 GO terms and 1 KEGG pathway (ribosome pathway) were enriched from the m^5^C-modified transcripts in the IVV (Fig. 1e). The enriched m^5^C on genes involved in the ribosome pathway in both no-infection and infection conditions indicate the essentiality of m^5^C methylations in regulating ribosome synthesis and assembly in the Ab-C98. Upon infection, the m^5^C methylations in the transcripts of energy production pathways were reduced. Similarly, in eukaryotes, m^5^C modification is associated with ribosome biogenesis and structure stability, and the loss of this methylation resulted in altered ribosome subunit assembly [29]. The m^5^C-modified CDS in the infection sample (IVV) was summarized in Table S3.

There are 31 m^5^C locations found only in the infection condition, mostly on the rRNA, followed by the CDS (Fig. 1f). The rRNA m^5^C found only in the IVV are the m^5^C97 and m^5^C1026 on one of the 16S rRNA copy (NNO94_02220), m^5^C350 on two 23S rRNA copies (NNO94_02235 and NNO94_14950), m^5^C2590 on five 23S rRNA copies (NNO94_00020, NNO94_02235, NNO94_14950, NNO94_17230 and NNO94_17500), m^5^C2757 on two 23S rRNA copies (NNO94_00020 and NNO94_02235) and m^5^C803 on one 23S rRNA (NNO94_17500) (Table S1). We noticed that these m^5^C sites were found in other rRNA copies in the IVB sample (Table S1). This indicates that the increased methylation ratio at these rRNA m^5^C regions in the infection conditions allowed the detection. The unique m^5^C-methylated CDS found in the infection conditions are the *exbD* (m^5^C102, m^5^C110) involved in iron transportation, *tig* (m^5^C269) involved in protein folding and *deaD* involved in RNA metabolism, containing four m^5^C locations on the coding region (m^5^C314, m^5^C1440, m^5^C1527 and m^5^C1755) (Table S3). The genes *atpG* and *rplP* involved in ATP synthesis and synthesis of large ribosomal protein L16, respectively, contain additional m^5^C methylations (m^5^C126 and m^5^C386, respectively) in the infection conditions compared to that in the no-infection conditions (Table S3). Interestingly, comparing these unique m^5^C-modified genes to the differentially expressed genes (DEGs) in Ab-C98 identified in our earlier study [12] found that the *exbD* and *deaD* genes were significantly upregulated upon the infection. Studies have reported the role of m^5^C methylations in regulating the mRNA gene expression in mammalian cells by affecting mRNA stability and translation efficiency [30,31]. Therefore, the unique presence of m^5^C methylations on the coding region of these mRNA potentially modulates the gene expression and bacterial survival in the host.

### Potential roles of m^6^A modification in regulating translation machinery in *A. baumannii* during infection

**Fig. 2.**
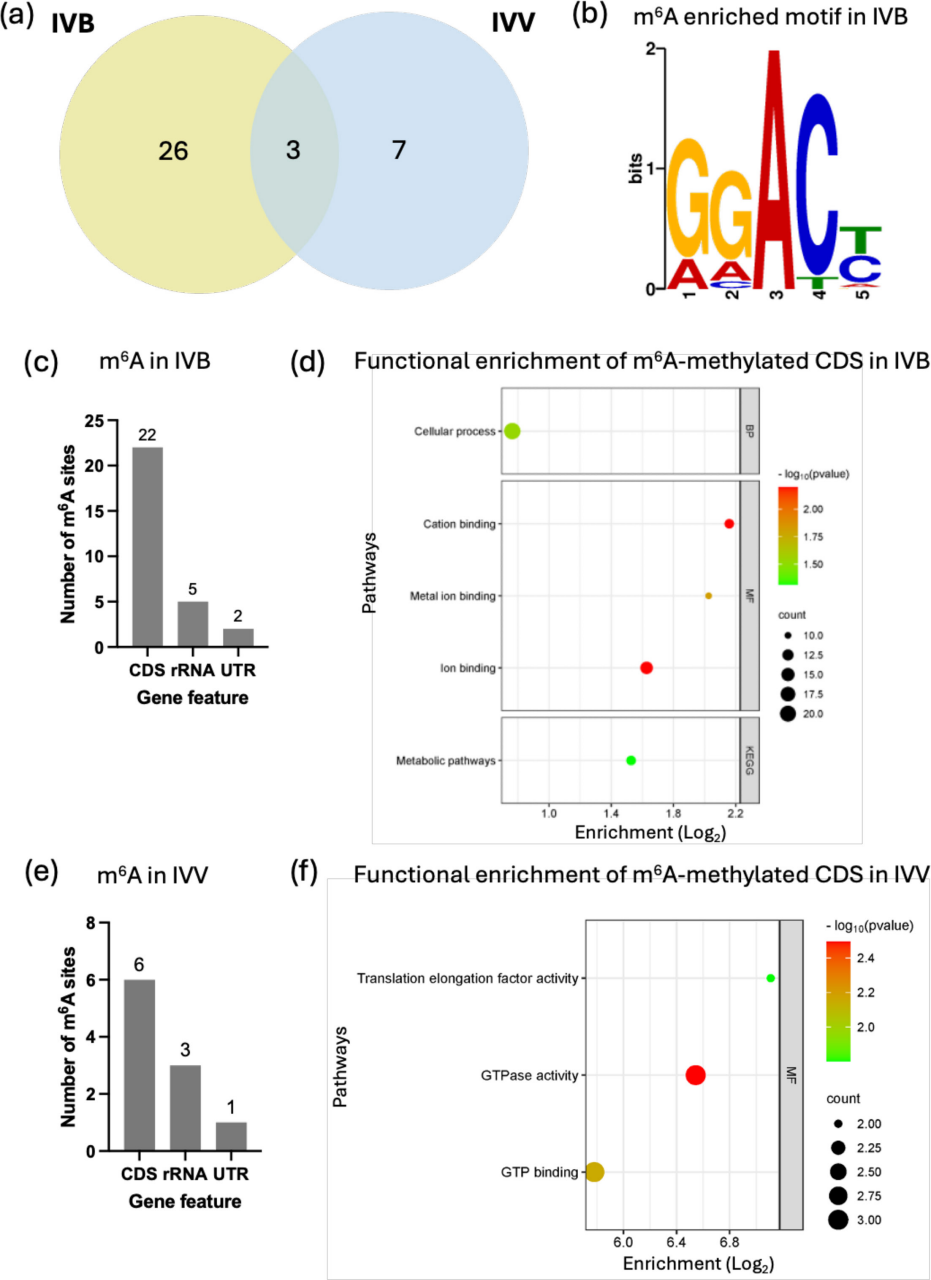
m^6^A modification analysis in Ab-C98 RNA. (**a**) Venn diagram of m^6^A predicted in the IVB and IVV Ab-C98. (**b**) *De novo* motif discovery of the m^6^A in Ab-C98 (IVB). (**c**) Gene features of the gene with predicted m^6^A in Ab-C98 no infection (IVB). (**d**) Functional enrichment analysis of the m^6^A-methylated CDS in Ab-C98 no infection (IVB). (**e**) Gene features of the gene with predicted m^6^A in Ab-C98 infection (IVV). (**f**) Functional enrichment analysis of the six m^6^A-methylated CDS in Ab-C98 infection (IVV). BP=Gene Ontology Biological process, MF=Gene Ontology Molecular Function, KEGG=Kyoto Encyclopaedia of Genes and Genomes.

m^6^A modification is well characterized in bacterial rRNA, playing critical roles in bacterial fitness and growth [1,32], but its presence and function in bacterial mRNA are still largely unknown. The m^6^A methylations on the Ab-C98 RNA were directly detected from the base-called reads via the bioinformatics tool m6Abasecaller. Twenty-nine m^6^A sites were predicted in the free-living Ab-C98 (IVB), while ten m^6^A sites were found in the infection Ab-C98 (IVV) (Fig. 2a). A unique motif sequence GGACY (E-value=2.7×10^−18^) was found on all 29 predicted m^6^A sites in the IVB sample (Fig. 2b); however, no enriched motif was found for the m^6^A in the IVV. This motif sequence differs from the reported m^6^A motif in *E. coli* and *Pseudomonas aeruginosa* mRNA (GCCAG) [33], suggesting that *A. baumannii* has a distinct different recognition site for the m^6^A methylase for methylation activities. On the Ab-C98 rRNA, m^6^A1513 was detected on four of the 16S rRNA copies, except one of the 16S rRNA (NNO94_00005) has two adjacent m^6^A detected (m^6^A1513 and m^6^A1514) (Table S1). No m^6^A was detected on the Ab-C98 23S rRNA by the m6Abasecaller tool.

Deng *et al.* [33] reported the high abundance of m^6^A in the mRNA of seven Gram-negative bacteria using the UHPLC-QQQ-MS/MS approach, and these m^6^A-modified transcripts were highly enriched in the energy and amino acids metabolism in *E. coli* and *P. aeruginosa*. Similarly, our analysis identified most of the m^6^A on the CDS of Ab-C98 in the IVB (Fig. 2c). All 21 m^6^A-modified CDS contain single m^6^A, except two m^6^A signals were detected within the ORF of *nuoN* gene encoding the NADH-quinone oxidoreductase subunit for energy production (Table S2). GO enrichment analysis demonstrated that these m^6^A-methylated genes were enriched in cation binding, metal-ion binding and ion binding (GO terms: MF), which are essential for numerous cellular processes (Fig. 2d). In the IVV, the m^6^A methylations were mostly annotated on six CDS (Fig. 2e) and were found uniquely in the infection sample. The m^6^A-methylated CDS involves the *feoB*, *exbD* and *basG* (which are genes involved in iron acquisition), RNA polymerase *rpoC*, elongation factor G for protein translation (*fusA*) and translational GTPase TypA (Table S3), in which significant upregulation of the *feoB*, *basG* and *exbD* was identified in our earlier study [12]. As iron acquisition plays essential roles in the virulence of *A. baumannii* [12,34], the functional roles of m^6^A on these iron uptake genes should be further investigated. Functional enrichment analysis of the six m^6^A-methylated CDS revealed significant enriched GTPase activity, GTP binding and translation elongation factor activity by at least 54.95-fold (Fig. 2f). This suggests that the host environment during the infection may have triggered an altered translational regulation in the bacterium, such as the production of virulence proteins, by increasing m^6^A methylations on the transcripts involved in these pathways and affecting the downstream gene expression.

### Pseudouridine and its putative regulatory effects on protein synthesis, metabolism and secondary metabolite biosynthesis in *A. baumannii*

**Fig. 3.**
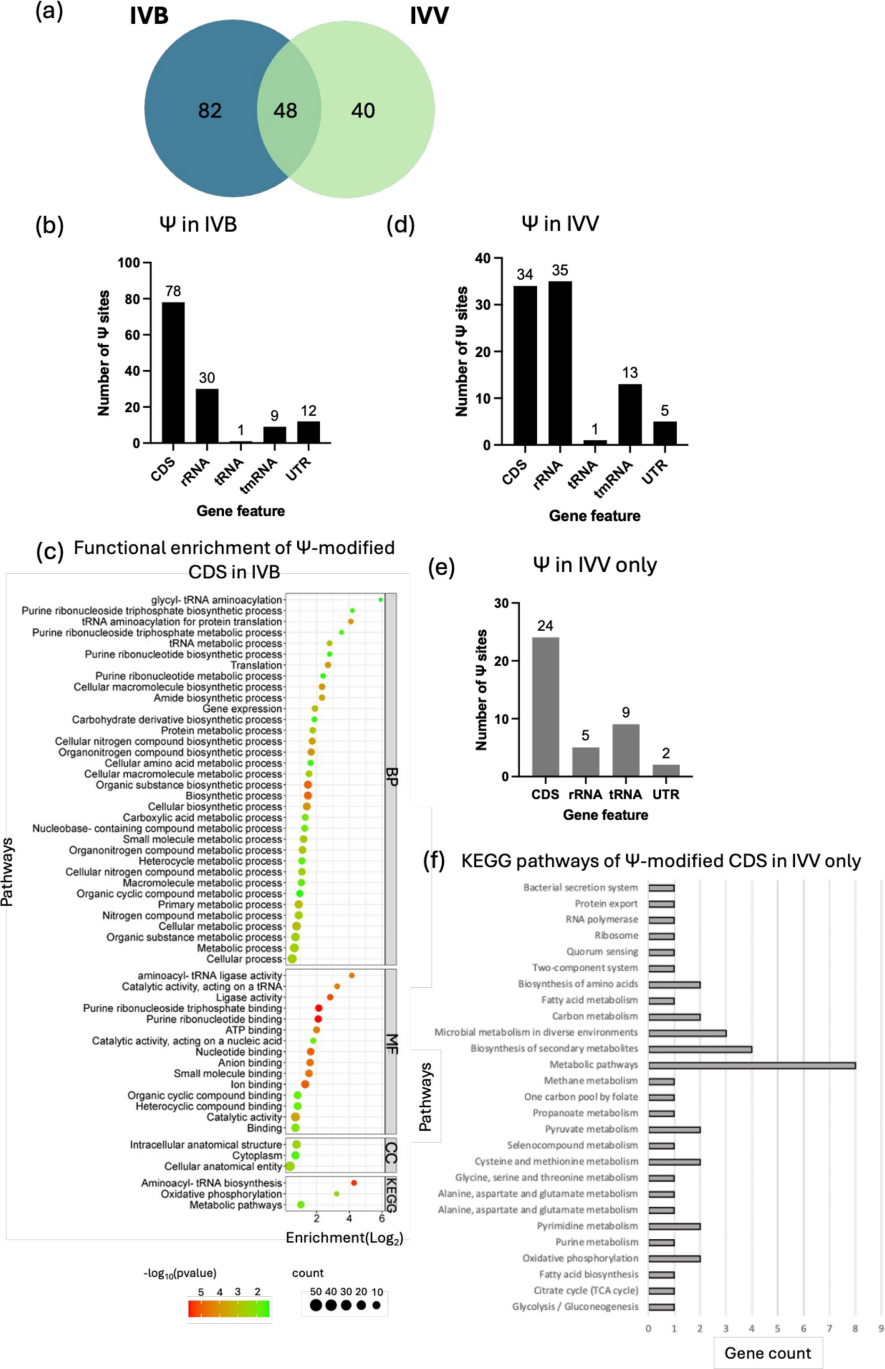
Ψ modifications in the Ab-C98 RNA. (**a**) Venn diagram of the Ψ present in the no-infection control (IVB) and the infection samples (IVV). (**b**) Gene features of the genes with predicted Ψ in Ab-C98 no infection (IVB). (**c**) Functional enrichment analysis of the Ψ-modified CDS in Ab-C98 no infection (IVB). BP=Gene Ontology Biological process, CC=Gene Ontology Cellular Components, MF=Gene Ontology Molecular Function, KEGG=Kyoto Encyclopaedia of Genes and Genomes. (**d**) Gene features of the genes with predicted Ψ in Ab-C98 infection (IVV). (**e**) Gene features of Ψs found uniquely in Ab-C98 infection (IVV only). (**f**) KEGG pathways annotation of the 20 Ψ-modified CDS presents only in the Ab-C98 infection (IVV).

The biological relevance of Ψ has been reported in regulating diverse biological functions of different RNA species. Although Ψ has been well-characterized in bacterial rRNA and tRNA, its presence on bacterial mRNA remains poorly explored due to the detection limits [35]. This study identified 130 ‘U’ sites as Ψ-modified in the Ab-C98 under no-infection conditions (IVB) (Fig. 3a), with a Ψ/U ratio of 0.09% in the whole transcriptome. This is lower than the reported Ψ ratio in *E. coli* mRNA (~12%) [36] and in mammalian mRNA (0.2–0.6%) [37], likely due to the lower coverage of our direct RNA-seq. Nevertheless, our analysis identified two Ψs (Ψ511 and Ψ783) on three of the six 16S rRNA copies encoded in the forward strand (Table S1). We noticed that the rRNA clusters encoded on the reverse strand had low coverage, thus failing the analysis threshold. The Ψ511 aligns with the reported *A. baumannii* Ψ511 in the MODOMICS database (rRNA ID: 1083) [38] and is located at a similar position to the Ψ516 reported in the *E. coli* 16S rRNA [39]. Eight Ψs were consistently detected on 3 of the 23S rRNA copies, which are Ψ945, Ψ1900, Ψ1906, Ψ2493, Ψ2541, Ψ2544, Ψ2569 and Ψ2594, except 1 of the 23S rRNA (NNO94_02235) had an additional Ψ2446 (Table S1). These Ψs are located at similar positions as the Ψs reported on *E. coli* 23S rRNA, indicating that these Ψs are conserved in bacteria. However, the differences in the numbering of Ψ on the rRNAs (corresponding to the nt position of the Ψ) might be due to the differences in the gene sequences between *A. baumannii* and *E. coli*.

In the IVB, Ab-C98 mRNA coding sequences had the most number of Ψ predicted, followed by rRNA and untranslated regions (Fig. 3b). Among the 68 Ψ-modified CDS, the *edd* gene encoding phosphogluconate dehydratase involved in the Entner–Doudoroff pathway for energy generation consists of four Ψ candidate sites, followed by the *abeM* gene (multidrug efflux MATE transporter) and NNO94_03250, encoding the beta-ketoacyl-ACP (Acyl carrier protein) synthase II for fatty acid production, with three Ψ locations (Table S2). Functional enrichment analysis revealed that 52 GO terms and 3 KEGG pathways were enriched (Fig. 3c). KEGG analysis indicated that the Ψ-modified genes were highly enriched in the aminoacyl-tRNA biosynthesis (19.50-fold), oxidative phosphorylation (9.33-fold) and metabolic pathways (2.04-fold) (Fig. 3c). The biosynthesis of aminoacyl-tRNA determines the accurate decoding of mRNA by precisely matching amino acids with tRNAs according to the genetic code, which is an ATP-demanding process [40]. Therefore, the high occurrence of Ψ involved in these pathways suggests their roles in the translation processes of this bacterium. Eighty-eight Ψ sites were found in the IVV, and most were found on the CDS and rRNA (Fig. 3d). A comparison of the Ψ-modified genes between the IVB and IVV found 40 Ψs present uniquely in the infection Ab-C98, and 24 are located on the CDS (Fig. 3e). These Ψ-modified genes are mostly involved in the metabolic pathways (*nrdA*, *metH*, *accB*, *pckG*, *carB*, *serA*, *atpB* and *atpC*) (Fig. 3f), which are critical for maintaining bacterial biological processes that are essential for bacterial viability and growth. Besides, 4 out of the 20 Ψ-modified genes are involved in the biosynthesis of secondary metabolites (*metH*, *accB*, *pckG* and *serA*) (Fig. 3f), whose products likely protected the bacterium from the harsh environment (host niche) and promoted bacterial adaptation and survival during the infection [41]. Similarly, significant upregulation was observed in the genes involved in the iron acquisition (*bfnL* and *feoB*) and the cold-shock protein *csp1*, according to our earlier study [12].

It has been reported that Ψ, located within ORFs of mRNAs, affects the translation speed and mRNA decoding, inducing the misincorporation of amino acids in the resulting protein [42,44], which might alter the protein function. Therefore, we conducted the SIFT analysis to predict the effect of Ψ located on the Ab-C98 CDS. SIFT analysis identified up to three deleterious amino acid substitutions for the genes with Ψ at the first and second positions of the codon (Table S4). In contrast, Ψ at the third position of the codon has minimum effect as 0 or 1 deleterious amino acid substitution was predicted as a single amino acid can be encoded by multiple codons (Table S4). To confirm the presence of Ψ predicted from the RNA modification analysis, we shortlisted Ψ-modified targets based on the SIFT analysis, which met the following criteria: (i) highest probability of Ψ modification in infection, while lowest probability of Ψ modification in the no-infection control; (ii) Ψ predicted at the first and second position of the codons, with the highest number of deleterious amino acid substitution predicted. This led to two Ψ mRNA targets: Ψ91 on the *atpB* gene and Ψ1280 on the *rpoC* gene.

**Fig. 4.**
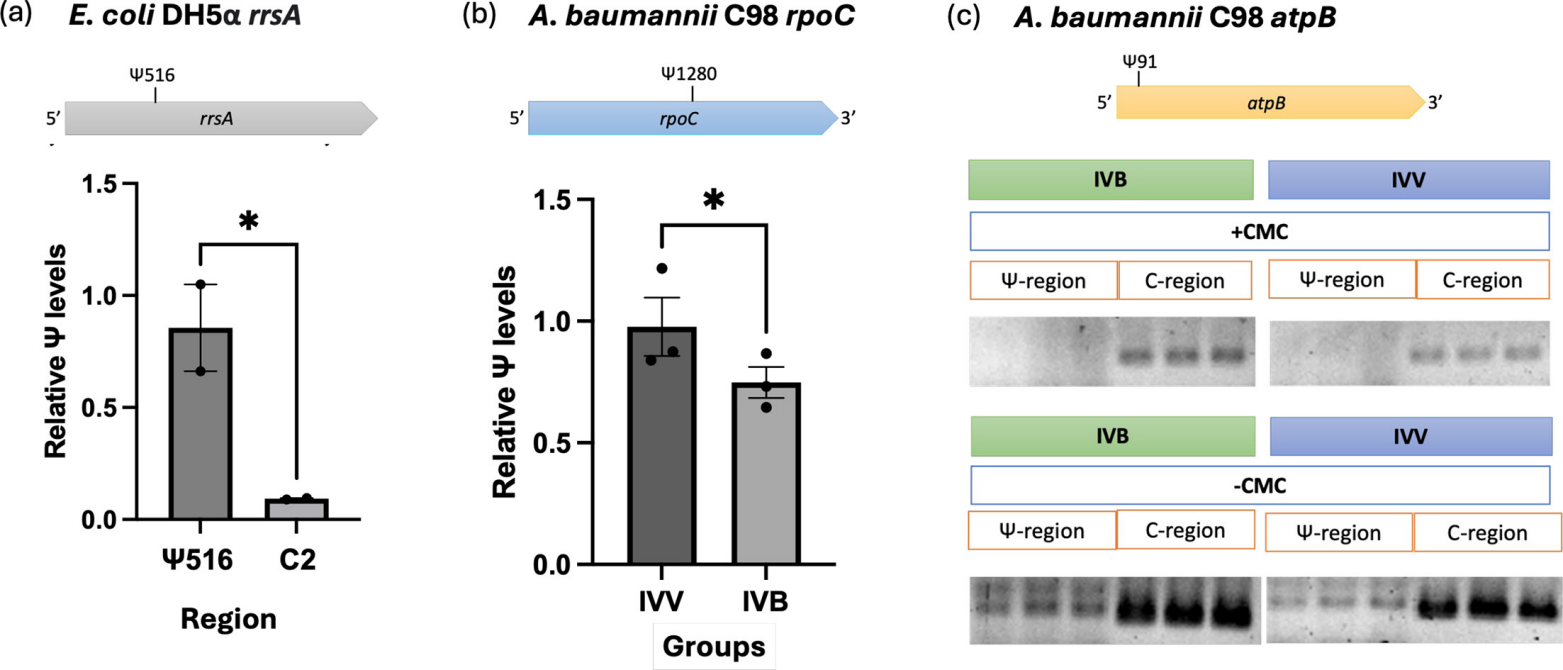
Detection of Ψ on targeted mRNA via CMC-assisted RT-qPCR. (**a**) Relative Ψ level of Ψ516 region on the *E. coli* DH5α *rrsA* 16S rRNA via CMC-assisted RT-qPCR. An additional Ψ-free control-region was used as a negative control (c2). (**b**) Relative Ψ level of Ψ1280 on *rpoC* mRNA in Ab-C98 infection (IVV) and no-infection control (IVB) via CMC-assisted RT-qPCR. A P-value <0.05 indicates a statistically significant difference in relative Ψ level between the targeted region or treatment groups. (**c**) Detection of Ψ91 on *atpB* mRNA in Ab-C98 infection (IVV) and no-infection control (IVB) via CMC-assisted RT semi-qPCR. The same volume of PCR products was loaded onto 2% agarose gel and visualized.

We used CMC-assisted RT-qPCR to validate the Ψ at the targeted sites independently. Upon the binding of CMC to the Ψ residue on the targeted RNA, CMC-Ψ adduct formed and blocked the reverse transcription. Thus, Ψ-modified RNA will produce no cDNA products and generate a higher Cq value in the qPCR than the non-Ψ-modified RNA [21]. The presence of Ψ was interpreted as a relative Ψ level compared to the control (non-modified region). Using Ψ516 on *E. coli rrsA* 16S rRNA as a control, we observed a significant increase in the relative Ψ level at the Ψ516 region compared to the control-region without any Ψ (C2) (*P*=0.03) (Fig. 4a). In Ab-C98, a higher Ψ level was detected in the *rpoC* mRNA in the IVV (*P*=0.04), compared to the IVB (Fig. 4b), consistent with our Ψ bioinformatics analysis. Studies have reported that amino acid substitution in *rpoC* resulted in a slower rate of transcriptional elongation [45]. Thus, the stressful environment of the host might have triggered an elevated Ψ of the *rpoC* mRNA in Ab-C98, resulting in an altered gene expression machinery in the pathogen to adapt to the host environment. Detection of Ψ91 on the *atpB* mRNA was performed by semi-qPCR due to the formation of strong primer dimers in the SYBR green-based qPCR. We successfully detected the presence of Ψ91 on *atpB* mRNA in the Ab-C98 under both IVV and IVB conditions, as indicated by the absence of PCR products due to blockage of CMC-Ψ adducts (Fig. 4c). The presence of Ψ91 on *atpB* in both conditions suggests the essentiality of Ψ on *atpB* mRNA in Ab-C98 for energy production to maintain bacterial cell viability.

## Conclusion

This is the first study reporting the transcriptome-wide m^5^C, m^6^A and Ψ modifications in *A. baumannii*. We reported the enriched modifications on the protein-coding genes and their potential roles in the biological pathways of the virulent community-acquired *A. baumannii* (Ab-C98), as well as during infection in *G. mellonella* larvae. Our findings were mainly based on high-throughput sequencing and bioinformatics analysis. Thus, further molecular experiments are required to confirm our observations. Nevertheless, our findings provide valuable references to the RNA modifications in *A. baumannii*. These could serve as preliminary findings for future research on novel drug discovery targeting these RNA modifications to combat this pathogen.

## supplementary material

10.1099/mgen.0.001327 Uncited Fig. S1.

## Abbreviations

CDS: coding sequence
CMC: carbodiimide *N*-cyclohexyl-*N*′-*β*-(4-methylmorpholino)ethylcarbodiimide *p*-toluenesulfonate
direct RNA-seq: direct RNA sequencing
IVB: no-infection controls
IVT: *in vitro* transcription control
IVV: infection samples
m^6^A: N^6^-methyladenosine
m^5^C: 5-methylcytosine
mRNA: messenger RNA
RNase_P_RNA: ribonuclease P RNA
rRNA: ribosomal RNA
tmRNA: transfer messenger RNA
tRNA: transfer RNA
Ψ: pseudouridine
