# Supplementary material for: Uncovering the transcriptome-wide RNA modifications in Acinetobacter baumannii
Source: Microb Genom. 2024 Nov 20;10(11):001327. doi: 10.1099/mgen.0.001327 (PMC11578064; doi:10.1099/mgen.0.001327)
Supplement: Uncited Fig. S1. [file mgen-10-01327-s001.pdf]

**Uncovering the transcriptome-wide RNA modifications in *Acinetobacter baumannii***

Kah Ern Ten[1], Sadequr Rahman[1], Hock Siew Tan[1*]

[1] School of Science, Monash University Malaysia, Bandar Sunway, Selangor Darul Ehsan, Malaysia

* Corresponding author
Email: tan.hocksiew@monash.edu (HST)

Table S1. Modified nucleotides on the rRNA of Ab-C98.

| **IVB** (no-infection) | | | | |
|---|---|---|---|---|
| Modifications | rRNA | Locus tag | Number of modified sites | Positions of modified nucleotides on the gene |
| $m^5C$ | 16S rRNA | NNO94_00005 | 6 | 97,931,1026,1054,1223,1434 |
| | | NNO94_02220 | 4 | 931,1054,1223,1434 |
| | | NNO94_14800 | 6 | 97,931,1026,1054,1223,1434 |
| | | NNO94_14965 | 5 | 97,931,1054,1223,1434 |
| | | NNO94_17245 | 5 | 97,931,1054,1223,1434 |
| | | NNO94_17485 | 5 | 97,931,1054,1223,1434 |
| | 23S rRNA | NNO94_00020 | 13 | 350,988,1149,1652,1743,1757,1789,2014,2073,2126,2289,2719,2859 |
| | | NNO94_02235 | 12 | 988,1149,1652,1743,1757,1789,2014,2073,2126,2289,2719,2859 |
| | | NNO94_14785 | 15 | 350,988,1149,1652,1743,1757,1789,2014,2073,2126,2289,2590,2719,2757,2859 |
| | | NNO94_14950 | 13 | 988,1149,1652,1743,1757,1789,2014,2073,2126,2289,2719,2757,2859 |
| | | NNO94_17230 | 14 | 350,988,1149,1652,1743,1757,1789,2014,2073,2126,2289,2719,2757,2859 |
| | | NNO94_17500 | 14 | 350,987,1148,1651,1742,1756,1788,2013,2072,2125,2288,2718,2756,2858 |
| $m^6A$ | 16S rRNA | NNO94_00005 | 2 | 1513,1514 |
| | | NNO94_02220 | 1 | 1513 |
| | | NNO94_14800 | 1 | 1513 |
| | | NNO94_14965 | 1 | 1513 |
| Ψ | 16S rRNA | NNO94_00005 | 2 | 511,783 |
| | | NNO94_02220 | 2 | 511,783 |
| | | NNO94_17485 | 1 | 783 |
| | 23S rRNA | NNO94_00020 | 8 | 945,1900,1906,2493,2541,2544,2569,2594 |
| | | NNO94_02235 | 9 | 945,1900,1906,2446,2493,2541,2544,2569,2594 |

| | | | | |
|---|---|---|---|---|
| | | NNO94_17500 | 8 | 944,1899,1905,2492,2540,2543,2568,2593 |
| **IVV** (infection) | | | | |
| Modifications | rRNA | Locus tag | Number of modified sites | Positions of modified nucleotides on the gene |
| $m^5C$ | 16S rRNA | NNO94_00005 | 5 | 97,931,1054,1223,1434 |
| | | NNO94_02220 | 6 | 97,931,1026,1054,1223,1434 |
| | | NNO94_17485 | 5 | 97,931,1054,1223,1434 |
| | | NNO94_14800 | 6 | 97,931,1026,1054,1223,1434 |
| | | NNO94_14965 | 5 | 97,931,1054,1223,1434 |
| | | NNO94_17245 | 5 | 97,931,1054,1223,1434 |
| | 23S rRNA | NNO94_00020 | 15 | 350,988,1149,1652,1743,1757,1789,2014,2073,2126,2289,2590,2719,2757,2859 |
| | | NNO94_02235 | 15 | 350,988,1149,1652,1743,1757,1789,2014,2073,2126,2289,2590,2719,2757,2859 |
| | | NNO94_17500 | 16 | 350,803,987,1148,1651,1742,1756,1788,2013,2072,2125,2288,2589,2718,2756,2858 |
| | | NNO94_14785 | 15 | 350,988,1149,1652,1743,1757,1789,2014,2073,2126,2289,2590,2719,2757,2859 |
| | | NNO94_14950 | 15 | 350,988,1149,1652,1743,1757,1789,2014,2073,2126,2289,2590,2719,2757,2859 |
| | | NNO94_17230 | 15 | 350,988,1149,1652,1743,1757,1789,2014,2073,2126,2289,2590,2719,2757,2859 |
| $m^6A$ | 16S rRNA | NNO94_14800 | 1 | 1513 |
| | | NNO94_14965 | 2 | 1514,1513 |
| Ψ | 16S rRNA | NNO94_00005 | 3 | 511,783,1189 |
| | | NNO94_02220 | 2 | 511,783 |
| | | NNO94_17485 | 2 | 511,783 |
| | 23S rRNA | NNO94_00020 | 9 | 945,1900,1906,2446,2493,2541,2544,2569,2594 |
| | | NNO94_02235 | 9 | 945,1900,1906,2446,2493,2541,2544,2569,2594 |

| | | | | |
|---|---|---|---|---|
| | | NNO94_17500 | 10 | 944,1899,1903,1905,2445,2492,2540,2543,2568,2593 |

*Modifications found uniquely in the IVV sample were underlined.

Table S2. Modified nucleotides on coding sequences of Ab-C98 in the free-living state (no-infection, IVB).

| Modifications | Gene name/Locus tag | Product | Number of modified sites | Positions of modified nucleotides on the gene |
|---|---|---|---|---|
| $m^5C$ | *tuf* | elongation factor Tu | 3 | 120,235,237 |
| | *rplK* | 50S ribosomal protein L11 | 2 | 251,389 |
| | *rplA* | 50S ribosomal protein L1 | 6 | 38,41,257,269,302,347 |
| | *rplJ* | 50S ribosomal protein L10 | 4 | 121,173,335,500 |
| | *rplL* | 50S ribosomal protein L7/L12 | 8 | 77,107,110,146,149,152,209,337 |
| | *rpoC* | DNA-directed RNA polymerase subunit beta' | 1 | 4109 |
| | *ompW* | outer membrane beta-barrel protein | 1 | 383 |
| | *rpsO* | 30S ribosomal protein S15 | 2 | 89,199 |
| | *rpmG* | 50S ribosomal protein L33 | 1 | 32 |
| | *tig* | trigger factor | 2 | 1088,1259 |
| | *ndk* | nucleoside-diphosphate kinase | 2 | 121,389 |
| | *secB* | protein-export chaperone SecB | 3 | 437,408,137 |
| | NNO94_01645 | enoyl-ACP reductase | 1 | 596 |
| | NNO94_01695 | ketol-acid reductoisomerase | 1 | 635 |
| | *rsfS* | ribosome silencing factor | 1 | 39 |
| | *infC* | translation initiation factor IF-3 | 2 | 149,317 |
| | *rpmI* | 50S ribosomal protein L35 | 1 | 164 |
| | *rplT* | 50S ribosomal protein L20 | 4 | 287,323,329,353 |

| | | | | |
|---|---|---|---|---|
| | NNO94_02115 | hypothetical protein | 12 | 245,242,239,197,194,182,179,149,137,50,32,29 |
| | *rpmF* | 50S ribosomal protein L32 | 1 | 164 |
| | *fabG* | 3-oxoacyl-ACP reductase FabG | 1 | 59 |
| | NNO94_03085 | ribose-phosphate pyrophosphokinase | 1 | 737 |
| | NNO94_03245 | protoporphyrinogen oxidase HemJ | 1 | 84 |
| | NNO94_03250 | beta-ketoacyl-ACP synthase II | 1 | 1022 |
| | *rpsL* | 30S ribosomal protein S12 | 1 | 340 |
| | *rpsG* | 30S ribosomal protein S7 | 1 | 373 |
| | *fusA* | elongation factor G | 6 | 626,674,677,680,1376,1583 |
| | *tuf* | elongation factor Tu | 5 | 89,120,123,235,237 |
| | *ahpC* | alkyl hydroperoxide reductase subunit C | 1 | 164 |
| | *tssC* | type VI secretion system contractile sheath large subunit | 2 | 845,1079 |
| | NNO94_05915 | type VI secretion system tube protein Hcp | 2 | 227,401 |
| | *rpsA* | 30S ribosomal protein S1 | 6 | 527,890,1103,1337,1340,1412 |
| | *rpsT* | 30S ribosomal protein S20 | 2 | 149,134 |
| | NNO94_07780 | HU family DNA-binding protein | 2 | 234,263 |
| | *eno* | phosphopyruvate hydratase | 2 | 371,235 |
| | *hfq* | RNA chaperone Hfq | 1 | 438 |
| | NNO94_10555 | bifunctional aconitate hydratase 2/2-methylisocitrate dehydratase | 2 | 2629,2183 |
| | *cyoA* | ubiquinol oxidase subunit II | 1 | 1028 |
| | *cyoB* | cytochrome o ubiquinol oxidase subunit I | 3 | 211,1778,1977 |
| | *cyoD* | cytochrome o ubiquinol oxidase subunit IV | 3 | 26,170,259 |
| | *cyoE* | heme o synthase | 2 | 83,84 |

| | | | | |
|---|---|---|---|---|
| | *rpsF* | 30S ribosomal protein S6 | 1 | 370 |
| | *rpsR* | 30S ribosomal protein S18 | 1 | 173 |
| | *rplI* | 50S ribosomal protein L9 | 4 | 137,188,191,194 |
| | *rpsU* | 30S ribosomal protein S21 | 2 | 89,152 |
| | NNO94_11260 | GatB/YqeY domain-containing protein | 2 | 145,251 |
| | *tsf* | translation elongation factor Ts | 5 | 854,581,335,305,293 |
| | *rpsB* | 30S ribosomal protein S2 | 3 | 407,391,176 |
| | NNO94_11740 | superoxide dismutase | 1 | 476 |
| | *efp* | elongation factor P | 4 | 284,194,191,49 |
| | NNO94_12365 | NADP-dependent isocitrate dehydrogenase | 2 | 2204,2084 |
| | NNO94_12675 | hypothetical protein | 2 | 80,392 |
| | NNO94_12935 | FAD-binding protein | 7 | 653,578,485,266,221,209,143 |
| | NNO94_13160 | phosphoenolpyruvate carboxykinase (GTP) | 1 | 887 |
| | *lpdA* | dihydrolipoyl dehydrogenase | 1 | 1379 |
| | *sdhA* | succinate dehydrogenase flavoprotein subunit | 2 | 1628,1477 |
| | *rplU* | 50S ribosomal protein L21 | 1 | 164 |
| | NNO94_14330 | OmpA family protein | 5 | 1093,1037,129,56,53 |
| | *rplM* | 50S ribosomal protein L13 | 3 | 39,401,403 |
| | *rpsI* | 30S ribosomal protein S9 | 2 | 62,263 |
| | NNO94_15000 | malate dehydrogenase | 3 | 533,410,121 |
| | *rplQ* | 50S ribosomal protein L17 | 3 | 371,276,218 |
| | *rpsD* | 30S ribosomal protein S4 | 3 | 557,337,286 |
| | *rpsK* | 30S ribosomal protein S11 | 1 | 197 |
| | *rpsM* | 30S ribosomal protein S13 | 2 | 104,44 |
| | *secY* | preprotein translocase subunit SecY | 4 | 833,760,758,434 |
| | *rplO* | 50S ribosomal protein L15 | 3 | 230,218,166 |

| | | | | |
|---|---|---|---|---|
| | *rpmD* | 50S ribosomal protein L30 | 1 | 45 |
| | *rpsE* | 30S ribosomal protein S5 | 3 | 347,194,170 |
| | *rplR* | 50S ribosomal protein L18 | 1 | 206 |
| | *rplF* | 50S ribosomal protein L6 | 2 | 290,182 |
| | *rplE* | 50S ribosomal protein L5 | 1 | 164 |
| | *rplX* | 50S ribosomal protein L24 | 2 | 305,182 |
| | *rpsQ* | 30S ribosomal protein S17 | 2 | 248,23 |
| | *rplP* | 50S ribosomal protein L16 | 3 | 365,362,146 |
| | NNO94_15270 | 30S ribosomal protein S3 | 2 | 191,100 |
| | *rplV* | 50S ribosomal protein L22 | 2 | 180,95 |
| | *rplD* | 50S ribosomal protein L4 | 6 | 575,572,569,326,257,50 |
| | *rplC* | 50S ribosomal protein L3 | 2 | 288,121 |
| | NNO94_15580 | Glu/Leu/Phe/Val dehydrogenase | 2 | 1208,695 |
| | *trmD* | tRNA (guanosine(37)-N1)-methyltransferase TrmD | 1 | 930 |
| | *rimM* | ribosome maturation factor RimM | 1 | 1051 |
| | NNO94_16385 | porin Omp33-36 | 3 | 182,873,874 |
| | NNO94_16535 | 2-oxo acid dehydrogenase subunit E2 | 1 | 572 |
| | *rpmH* | 50S ribosomal protein L34 | 1 | 123 |
| | *tviB* | Vi polysaccharide biosynthesis UDP-N-acetylglucosamine C-6 dehydrogenase TviB | 2 | 593,1151 |
| | NNO94_17735 | DegT/DnrJ/EryC1/StrS aminotransferase family protein | 1 | 760 |
| | *atpB* | F0F1 ATP synthase subunit A | 1 | 8 |
| | NNO94_18150 | F0F1 ATP synthase subunit B | 1 | 374 |
| | NNO94_18155 | F0F1 ATP synthase subunit delta | 1 | 298 |
| | *atpA* | F0F1 ATP synthase subunit alpha | 2 | 677,920 |
| | *atpG* | F0F1 ATP synthase subunit gamma | 5 | 83,119,358,775,821 |
| | *atpD* | F0F1 ATP synthase subunit beta | 2 | 359,1235 |

| | | | | |
|---|---|---|---|---|
| | NNO94_18175 | F0F1 ATP synthase subunit epsilon | 1 | 344 |
| $m^6A$ | NNO94_00150 | OprD family porin | 1 | 234 |
| | *infB* | translation initiation factor IF-2 | 1 | 133 |
| | *pnp* | polyribonucleotide nucleotidyltransferase | 1 | 1523 |
| | *clpX* | ATP-dependent Clp protease ATP-binding subunit ClpX | 1 | 756 |
| | NNO94_01580 | DUF4115 domain-containing protein | 1 | 469 |
| | *bamB* | outer membrane protein assembly factor BamB | 1 | 447 |
| | NNO94_02660 | ribonucleoside-diphosphate reductase subunit alpha | 1 | 1082 |
| | *nuoG* | NADH-quinone oxidoreductase subunit NuoG | 1 | 1232 |
| | *nuoN* | NADH-quinone oxidoreductase subunit NuoN | 2 | 304,1091 |
| | NNO94_05100 | adenylosuccinate synthase | 1 | 1277 |
| | NNO94_05900 | hypothetical protein | 1 | 267 |
| | *metK* | methionine adenosyltransferase | 1 | 460 |
| | *tkt* | transketolase | 1 | 844 |
| | NNO94_10240 | universal stress protein | 1 | 128 |
| | NNO94_11720 | NADP-dependent malic enzyme | 1 | 935 |
| | NNO94_12365 | NADP-dependent isocitrate dehydrogenase | 1 | 2151 |
| | *carB* | carbamoyl-phosphate synthase large subunit | 1 | 2077 |
| | NNO94_13355 | 2-oxoglutarate dehydrogenase E1 component | 1 | 2012 |
| | *typA* | translational GTPase TypA | 1 | 1700 |
| | NNO94_14360 | nitrite/sulfite reductase | 1 | 145 |
| | *secF* | protein translocase subunit SecF | 1 | 736 |
| Ψ | *feoB* | ferrous iron transporter B | 2 | 590,837 |
| | *tuf* | elongation factor Tu | 1 | 509 |
| | NNO94_00610 | hypothetical protein | 1 | 825 |
| | *tpiA* | triose-phosphate isomerase | 1 | 406 |
| | *infB* | translation initiation factor IF-2 | 1 | 1269 |

| | | | | |
|---|---|---|---|---|
| | *gspG* | type II secretion system major pseudopilin GspG | 1 | 482 |
| | *topA* | type I DNA topoisomerase | 1 | 993 |
| | *exbD* | biopolymer transporter ExbD | 1 | 191 |
| | *clpX* | ATP-dependent Clp protease ATP-binding subunit ClpX | 1 | 730 |
| | *edd* | phosphogluconate dehydratase | 4 | 562,660,62,756 |
| | *hisS* | histidine--tRNA ligase | 2 | 269,946 |
| | *bamB* | outer membrane protein assembly factor BamB | 1 | 756 |
| | NNO94_01645 | enoyl-ACP reductase | 1 | 66 |
| | *panC* | pantoate--beta-alanine ligase | 1 | 170 |
| | NNO94_02040 | ribosome-associated protein | 1 | 99 |
| | *pheT* | phenylalanine--tRNA ligase subunit beta | 1 | 1086 |
| | NNO94_02520 | methionine synthase | 1 | 334 |
| | *nuoC* | NADH-quinone oxidoreductase subunit C/D | 1 | 1543 |
| | *nuoG* | NADH-quinone oxidoreductase subunit NuoG | 1 | 473 |
| | NNO94_04460 | hypothetical protein | 1 | 96 |
| | NNO94_04465 | hypothetical protein | 1 | 37 |
| | NNO94_05980 | type IVB secretion system protein IcmH/DotU | 1 | 66 |
| | NNO94_09575 | hypothetical protein | 1 | 138 |
| | *recA* | recombinase RecA | 1 | 960 |
| | *cyoA* | ubiquinol oxidase subunit II | 1 | 529 |
| | *cyoE* | heme o synthase | 1 | 579 |
| | *rplI* | 50S ribosomal protein L9 | 1 | 43 |
| | *fis* | DNA-binding transcriptional regulator Fis | 1 | 42 |
| | *yegQ* | tRNA 5-hydroxyuridine modification protein YegQ | 1 | 423 |
| | *lipA* | lipoyl synthase | 1 | 877 |
| | NNO94_11720 | NADP-dependent malic enzyme | 1 | 1170 |
| | *dacC* | D-alanyl-D-alanine carboxypeptidase PBP5/6 | 2 | 267,332 |

| | | | | |
|---|---|---|---|---|
| | *purB* | adenylosuccinate lyase | 1 | 874 |
| | NNO94_12510 | pyridoxal phosphate-dependent aminotransferase | 1 | 543 |
| | *queC* | 7-cyano-7-deazaguanine synthase QueC | 1 | 260 |
| | *purM* | phosphoribosylformylglycinamidine cyclo-ligase | 1 | 850 |
| | *htpX* | protease HtpX | 1 | 67 |
| | NNO94_13160 | phosphoenolpyruvate carboxykinase (GTP) | 1 | 487 |
| | NNO94_13180 | hypothetical protein | 1 | 258 |
| | NNO94_13550 | valine--tRNA ligase | 1 | 2129 |
| | NNO94_13965 | outer membrane protein transport protein | 1 | 940 |
| | *gatA* | Asp-tRNA(Asn)/Glu-tRNA(Gln) amidotransferase subunit GatA | 1 | 986 |
| | NNO94_14220 | proline--tRNA ligase | 1 | 1319 |
| | *aspS* | aspartate--tRNA ligase | 1 | 69 |
| | *miaB* | tRNA (N6-isopentenyl adenosine(37)-C2)-methylthiotransferase MiaB | 1 | 1282 |
| | NNO94_15020 | PhoH family protein | 1 | 68 |
| | *glyQ* | glycine--tRNA ligase subunit alpha | 1 | 36 |
| | *glyS* | glycine--tRNA ligase subunit beta | 1 | 1541 |
| | NNO94_15510 | DUF262 domain-containing protein | 1 | 276 |
| | *hisA* | 1-(5-phosphoribosyl)-5-[(5-phosphoribosylamino)methylideneamino]imidazole-4-carboxamide isomerase | 1 | 404 |
| | NNO94_16260 | MFS transporter | 1 | 1142 |
| | *yidC* | | 1 | 1719 |
| | NNO94_17720 | glycosyltransferase family 4 protein | 1 | 164 |
| | NNO94_17730 | acetyltransferase | 1 | 442 |
| | NNO94_17735 | DegT/DnrJ/EryC1/StrS aminotransferase family protein | 1 | 1158 |
| | NNO94_17885 | D-amino acid dehydrogenase | 1 | 615 |

| | | | | |
|---|---|---|---|---|
| | NNO94_17895 | RidA family protein | 1 | 159 |
| | NNO94_18155 | F0F1 ATP synthase subunit delta | 1 | 388 |
| | *atpD* | F0F1 ATP synthase subunit beta | 1 | 531 |
| | NNO94_00945 | CBS domain-containing protein | 1 | 812 |
| | NNO94_01025 | AarF/UbiB family protein | 1 | 707 |
| | *abeM* | multidrug efflux MATE transporter AbeM | 3 | 1338,675,527 |
| | *pth* | aminoacyl-tRNA hydrolase | 1 | 146 |
| | NNO94_03250 | beta-ketoacyl-ACP synthase II | 3 | 777,771,231 |
| | NNO94_03410 | PilT/PilU family type 4a pilus ATPase | 1 | 1062 |
| | *ttcA* | tRNA 2-thiocytidine(32) synthetase TtcA | 1 | 407 |
| | NNO94_16645 | ankyrin repeat domain-containing protein | 1 | 47 |
| | NNO94_18245 | DUF2236 domain-containing protein | 1 | 787 |

Table S3. Modified nucleotides on coding sequences of Ab-C98 in the infection condition (IVV).

| Modifications | Gene name/Locus tag | Product | Number of modified sites | Positions of modified nucleotides on the gene |
|---|---|---|---|---|
| $m^5C$ | *tuf* | elongation factor Tu | 1 | 235 |
| | *rplK* | 50S ribosomal protein L11 | 2 | 251,389 |
| | *rplA* | 50S ribosomal protein L1 | 1 | 269 |
| | *rplJ* | 50S ribosomal protein L10 | 3 | 121,173,500 |
| | *rplL* | 50S ribosomal protein L7/L12 | 5 | 107,110,152,209,337 |
| | *rpsO* | 30S ribosomal protein S15 | 1 | 89 |
| | *exbD* | biopolymer transporter ExbD | 2 | 102,110 |
| | *tig* | trigger factor | 1 | 269 |
| | *ndk* | nucleoside-diphosphate kinase | 1 | 389 |
| | *rplT* | 50S ribosomal protein L20 | 3 | 287,329,353 |
| | NNO94_02115 | hypothetical protein | 6 | 242,194,179,149,50,29 |
| | *tuf* | elongation factor Tu | 3 | 123,235,237 |
| | *rpsA* | 30S ribosomal protein S1 | 1 | 1340 |
| | *rpsT* | 30S ribosomal protein S20 | 2 | 149,134 |
| | NNO94_10765 | cytochrome o ubiquinol oxidase subunit IV | 1 | 26 |
| | *rpsR* | 30S ribosomal protein S18 | 1 | 173 |
| | *rplI* | 50S ribosomal protein L9 | 4 | 137,188,191,194 |
| | *rpsU* | 30S ribosomal protein S21 | 2 | 89,152 |
| | NNO94_11260 | GatB/YqeY domain-containing protein | 1 | 145 |
| | NNO94_14330 | OmpA family protein | 5 | 1037,129,56,53,34 |
| | *rplM* | 50S ribosomal protein L13 | 1 | 39 |
| | *rpsI* | 30S ribosomal protein S9 | 1 | 62 |
| | *rplQ* | 50S ribosomal protein L17 | 2 | 371,276 |

| | | | | |
|---|---|---|---|---|
| | *rpsK* | 30S ribosomal protein S11 | 1 | 197 |
| | *rpsM* | 30S ribosomal protein S13 | 1 | 104 |
| | *rplO* | 50S ribosomal protein L15 | 1 | 166 |
| | *rpmD* | 50S ribosomal protein L30 | 1 | 45 |
| | *rpsE* | 30S ribosomal protein S5 | 1 | 347 |
| | *rpsQ* | 30S ribosomal protein S17 | 2 | 248,23 |
| | *rplP* | 50S ribosomal protein L16 | 3 | 386,365,362 |
| | *rplV* | 50S ribosomal protein L22 | 1 | 95 |
| | *rplD* | 50S ribosomal protein L4 | 4 | 575,572,569,326 |
| | *deaD* | DEAD/DEAH box helicase | 4 | 1755,1527,1440,314 |
| | *omp33-36* | porin Omp33-36 | 1 | 182 |
| | *atpG* | F0F1 ATP synthase subunit gamma | 2 | 126,358 |
| | *atpD* | F0F1 ATP synthase subunit beta | 1 | 1235 |
| $m^6A$ | *feoB* | ferrous iron transporter B | 1 | 1173 |
| | *rpoC* | DNA-directed RNA polymerase subunit beta | 1 | 1228 |
| | *exbD* | biopolymer transporter ExbD | 1 | 372 |
| | *fusA* | elongation factor G | 1 | 273 |
| | *basG* | acinetobactin biosynthesis histidine decarboxylase BasG | 1 | 164 |
| | *typA* | translational GTPase TypA | 1 | 1541 |
| Ψ | *feoB* | ferrous iron transporter B | 1 | 275 |
| | *secE* | preprotein translocase subunit SecE | 1 | 356 |
| | *rpoC* | DNA-directed RNA polymerase subunit beta' | 2 | 1280,1341 |
| | *infB* | translation initiation factor IF-2 | 1 | 1269 |
| | NNO94_05350 | transposase | 1 | 153 |
| | *csp1* | cold-shock protein | 1 | 153 |

| | | | | |
|---|---|---|---|---|
| | *bfnL* | acetyltransferase | 1 | 321 |
| | *accB* | acetyl-CoA carboxylase biotin carboxyl carrier protein | 1 | 111 |
| | *pckG* | phosphoenolpyruvate carboxykinase (GTP) | 1 | 1707 |
| | *carB* | carbamoyl-phosphate synthase large subunit | 2 | 1307,1957 |
| | *serA* | phosphoglycerate dehydrogenase | 1 | 170 |
| | *omp33-36* | porin Omp33-36 | 2 | 449,860 |
| | *atpB* | F0F1 ATP synthase subunit A | 2 | 91,629 |
| | *atpH* | F0F1 ATP synthase subunit delta | 1 | 388 |
| | *atpC* | F0F1 ATP synthase subunit epsilon | 1 | 65 |
| | NNO94_01025 | AarF/UbiB family protein | 1 | 707 |
| | *abeM* | multidrug efflux MATE transporter AbeM | 1 | 675 |
| | *nrdA* | ribonucleoside-diphosphate reductase subunit alpha | 1 | 1482 |
| | *pth* | aminoacyl-tRNA hydrolase | 2 | 162,146 |
| | *rplY* | 50S ribosomal protein L25 | 1 | 295 |
| | *fabB_1* | beta-ketoacyl-ACP synthase II | 3 | 777,771,231 |
| | NNO94_03410 | PilT/PilU family type 4a pilus ATPase | 1 | 791 |
| | *metH* | methionine synthase | 1 | 3550 |
| | *ychF* | redox-regulated ATPase YchF | 1 | 1074 |
| | NNO94_10940 | HAMP domain-containing histidine kinase | 1 | 1039 |
| | NNO94_16645 | ankyrin repeat domain-containing protein | 1 | 47 |
| | NNO94_18245 | DUF2236 domain-containing protein | 1 | 787 |

*Modifications found uniquely in the IVV sample were underlined.

Table S4. SIFT analysis of the 20 Ψ-modified mRNA in Ab-C98 infection sample.

| Ψ located on codon | Locus tag | Ψ position in the sequence | Probability of Ψ modification in infection | Probability of Ψ modification in no infection control | Gene product/ gene name | Number of deleterious amino acid substitution | Deleterious amino acid substitution |
|---|---|---|---|---|---|---|---|
| 1 | NNO94_18140 | 91 | 0.96 | 0.425 | *atpB* | 2 | W31R, W31G |
| | NNO94_03890 | 3550 | 0.965 | 0.79 | *metH* | 0 | - |
| | NNO94_13260 | 1957 | 0.995 | 0.8 | *carB* | 2 | L653I, L653V |
| | NNO95_03080 | 295 | 0.97 | 0.85 | *rplY* | 0 | (may suppress transcriptional stop) |
| | NNO94_10940 | 1039 | 0.95 | N/A | HAMP domain-containing histidine kinase | 3 | Y347D, Y347H, Y347N |
| 2 | NNO94_00520 | 1280 | 0.92 | 0.155 | *rpoC* | 3 | M427R, M427T, M427K |
| | NNO94_18140 | 629 | 0.93 | 0.265 | *atpB* | 2 | I210S, I210N |
| | NNO94_00485 | 356 | 0.92 | 0.29 | *secE* | 2 | V119G, V119A |
| | NNO94_16385 | 449 | 0.935 | 0.315 | *omp33-36* | N/A | N/A |
| | NNO94_13260 | 1307 | 0.91 | 0.505 | *carB* | 3 | I436S, I436N, I436T |
| | NNO94_16385 | 860 | 0.92 | 0.61 | *omp33-36* | N/A | N/A |
| | NNO94_15665 | 170 | 0.975 | 0.615 | *serA* | 0 | - |
| | NNO94_18175 | 65 | 0.905 | 0.795 | F0F1 ATP synthase subunit epsilon | 0 | - |
| | NNO94_00305 | 275 | 0.935 | 0.85 | *feoB* | 2 | F92S, F92C |
| | NNO94_03410 | 791 | 0.92 | 0.89 | PilT/PilU family type 4a pilus ATPase | 2 | M264K, M264R |
| 3 | NNO94_10070 | 111 | 0.94 | 0.06 | *accB* | 0 | - |
| | NNO94_00520 | 1341 | 0.97 | 0.195 | *rpoC* | 1 | I447M |

| | | | | | | | |
|---|---|---|---|---|---|---|---|
| | NNO94_13160 | 1707 | 0.905 | 0.35 | phosphoenolpyruvate carboxykinase (GTP) | 0 | - |
| | NNO94_02660 | 1482 | 0.91 | 0.35 | ribonucleoside-diphosphate reductase subunit alpha | 0 | (may induce premature stop) |
| | NNO94_03075 | 162 | 0.91 | 0.855 | *pth* | 0 | - |
| | NNO94_07880 | 321 | 0.935 | N/A | acetyltransferase | 0 | - |
| | NNO94_05375 | 153 | 0.925 | N/A | cold-shock protein | 1 | F51L |
| | NNO94_07075 | 1074 | 0.925 | N/A | *ychF* | 0 | - |
| | NNO94_05350 | 153 | 0.95 | N/A | transposase | 0 | - |
